# Development of a Monoclonal Antibody-Based Sandwich ELISA for Peanut Allergen Ara h 1 in Food

**DOI:** 10.3390/ijerph10072897

**Published:** 2013-07-12

**Authors:** Juan Peng, Shanshan Song, Liguang Xu, Wei Ma, Liqiang Liu, Hua Kuang, Chuanlai Xu

**Affiliations:** State Key Laboratory of Food Science & Technology, School of Food Science & Technology, Jiangnan University, Wuxi 214122, China; E-Mails: pengjuan2016@163.com (J.P.); sss@jiangnan.edu.cn (S.S.); xuliguang2006@yahoo.com.cn (L.X.); mawei209@126.com (W.M.); raxray@gmail.com (L.L.); xcl@jiangnan.edu.cn (C.X.)

**Keywords:** peanut allergen, Ara h 1, monoclonal antibody, sandwich ELISA

## Abstract

We have established a highly sensitive sandwich enzyme-linked immunosorbent assay (ELISA) based on two monoclonal antibodies (mAb) to measure the content of the major peanut allergen Ara h 1 in foods. Two mAbs were selected out of 12 murine hybridoma cells secreting Ara h 1-specific antibody. Using mAb 6 as the capture antibody and HRP-labelled mAb 4 as the detection antibody, the limit of detection (LOD) the assay was 0.34 ng/mL. Cross-reaction analysis showed that this method was strongly specific and had no cross-reactions with Ara h 2, pea protein or soy protein. Sample analysis showed that this ELISA was a useful tool to monitor peanut allergens in food products by measuring Ara h 1 content.

## 1. Introduction

The peanut (*Arachis hypogaea* L.) is a common food material and one of the most frequent causes of food allergies, accounting for approximately one-third of all severe allergic reactions [1,2]. Peanut allergies affect approximately 0.5%–0.7% of children and can be a lifelong affliction in most cases [3,4]. Very low amounts (~100 µg) of peanut protein are sufficient to elicit mild reactions in peanut-sensitized persons [5,6]. Consequently, strict avoidance of peanut-containing foods is the only possibility to prevent allergic reaction for consumers with peanut allergies [7]. To prevent peanut-sensitized persons from unintentional ingestion of peanut allergens, existing food labeling practices have been modified by food manufacturers to identify the presence of important food allergens in their products [8]. In addition, a sensitive analytical method to detect hidden allergens in foods is essential.

Sensitization in up to 95% of peanut-allergic patients has been attributed to Ara h 1, a 65-kDa glycoprotein which comprises 12%–16% of the total protein content in peanut extracts and is an established major food allergen [9,10]. The stable trimeric structure of Ara h 1 prevents IgE binding epitopes from degradation, thereby preserving allergenicity of peanuts during food processing [11,12]. Therefore, Ara h 1 presents an effective marker to monitor peanut allergen content in food products.

The most commonly used analytical method for allergen detection is based on the enzyme-linked immunosorbent assay (ELISA) technique owing to its high sensitivity and specificity without the need for sophisticated equipment [13,14,15]. Here, we report the development of a mAb-based sandwich ELISA to monitor content of the peanut allergen Ara h 1 in foods by comparing sequential Ara h 1 levels. Although a monoclonal antibody-based ELISA has been established to measure Ara h 1 in foods, the present study will develop a highly sensitive and more convenient sandwich ELISA [10].

## 2. Experimental Section

### 2.1. Materials

An Ara h 1 standard (ST-AH1) was purchased from INDOOR Biotechnologies, Inc. (Charlottesville, VA, USA). HAT supplement (containing hypoxanthine, aminopterin and thymidine; 50×), HT supplement (containing hypoxanthine and thymidine; 100×), polyethylene glycol 1450, complete and incomplete Freund’s adjuvant, and goat anti-mouse immunoglobulin (Ig)G antibody were purchased from Sigma-Aldrich (St. Louis, MO, USA). Fetal bovine serum albumin (BSA) and Roswell Park Memorial Institute 1640 medium were obtained from Sunshine Biotechnology Co., Ltd. (Nanjing, China). 3,3′,5,5′-Tetramethylbenzidine (TMB) substrate and horseradish peroxidase (HRP) were purchased from Aladdin Chemistry Co., Ltd. (Shanghai, China). Seven types of processed foods containing peanut products and three types with no declaration regarding the presence of peanut or peanut components in the list of ingredients were purchased from the Wangvard Market in Wuxi, China. All other reagents and chemicals were purchased from the National Pharmaceutical Group Chemical Reagent Co., Ltd. (Beijing, China). Eight-week-old female BABL/c mice were purchased from the Shanghai Laboratory Animal Center (Shanghai, China).

### 2.2. Ara h 1 Purification

Fresh peanuts (10 g) were ground and then defatted by shaking in petroleum ether (100 mL) for 4 h at 4 °C in a water bath, which was repeated three times, then the mixture centrifuged at 8,000 rpm for 10 min and the protein content from the supernatant was extracted using 0.01 M phosphate-buffered saline (PBS, 100 mL) overnight at 4 °C in a water bath while shaking. After centrifugation at 8,000 rpm for 10 min, crude protein extract was obtained. The Ara h 1 protein was then purified via ammonium sulfate precipitation and cation exchange chromatography [11].

### 2.3. Ara h 1 mAb Preparation

Ara h 1-specific mAbs were obtained using a standard protocol [16]. Five female BALA/c mice were subcutaneously injected with Ara h 1 (100 µg) at 21 day intervals. After 3 months, the mouse with the highest titer was intraperitoneally injected with Ara h 1 (30 µg). Three days later hybridoma cells were formed through the fusion of splenocytes and Sp2/0 murine myeloma cells (Chinese Academy of Sciences, Shanghai, China). The positive cells were selected by indirect ELISA and then subcloned three times by limiting dilutions. In this experiment, 12 cell strains were obtained and mAbs accordingly were obtained from the ascites of mice injected intraperitoneally with screened hybridoma cells and purified using the caprylic acid-ammonium sulfate precipitation method.

### 2.4. HRP-Labeled mAbs

First, HRP was mixed with 0.06 M NaIO_4_ for 30 min at 4 °C and then 0.16 M glycol was added to the mixture at room temperature. After 30 min, purified mAbs were added to the mixture and a pH of 9.0 was maintained by the addition of 0.05 M carbonate buffer (pH 10.0). After 16 h, the reaction was terminated by the addition of NaBH_4_ solution. An equal volume of saturated (NH_4_)_2_SO_4_ solution was added to obtain HRP-conjugated antibodies, which were dissolved in 0.01 M PBS to a concentration of 2 mg/mL and characterized by direct ELISA. Importantly, all the solutions must be stored in the dark.

### 2.5. Indirect ELISA

The anti-Ara h 1 activity in serum titers and hybridoma cell lines was detected using an indirect ELISA. Briefly, ST-AH1 in coating buffer (0.01 M phosphate buffered saline, pH 9.6, 100 μL of 0.2 µg/mL solution) was added to each well of a 96-well microplate and incubated overnight at 37 °C. After washing three times with washing buffer (0.01 M PBS (pH 7.4) containing 0.05% (v/v) Tween 20), the ELISA plates were blocked with blocking buffer (coating buffer containing 1% (w/v) gelatin) (200 μL/well) for 2 h at 37 °C. After washing, cell supernatant or mouse serum, serially diluted with antibody dilution buffer (PBS containing 0. 1% (w/v) gelatin and 0.05% (v/v) Tween 20), was added to the wells (100 μL/well) and incubated for 30 min at 37 °C. After washing, HRP-labeled goat anti-mouse immunoglobulin (100 μL), diluted with antibody dilution buffer at a ratio of 1:3,000, was added to each well and the plate was incubated for 30 min at 37 °C. After washing six times, the colorimetric assay was developed by the addition of 100 μL of TMB substrate solution. After 15 min, the reaction was stopped by the addition of 2 M sulfuric acid (50 μL/well) and the protein concentrations were determined using a spectrophotometer at an optical density (OD) of 450 nm (OD_450_).

### 2.6. Sandwich ELISA

Each well of a 96-well microplate was coated with coating buffer (100 µL) containing 2 µg/mL of anti-Ara h 1 mAb and incubated overnight at 4 °C. Then, the wells were washed three times with 250 mL/well of washing buffer, and 200 μL/well of blocking buffer was added and incubated for 2 h at 37 °C. After washing three times, 100 μL of serially diluted ST-AH1 or sample extract solution was added to the corresponding wells and incubated for 1 h at 37 °C. After washing the plates, 100 µL of HRP-labeled anti-Arah1 mAb was added to each well and then the microplate was incubated for 1 h at 37 °C. After washing six times, 100 mL/well of freshly prepared TMB substrate solution was added and reacted for 15 min at 37 °C in the dark. The reaction was stopped by the addition of 2 M sulfuric acid (50 μL/well) and the absorbance was measured at OD_450_ using a microplate reader. All measurements were made in triplicate.

### 2.7. Pairwise Interaction Analysis

According to the sandwich ELISA protocol, the optimal mAb combination was obtained by using anti-Ara h 1 mAb at 2 µg/well as the capture mAb, and the other 11 HRP-labelled mAbs at a dilution of 1:800 for detection. A positive control of 200 ng/mL of ST-AH1 in PBS and negative control of 0.01 M PBS were added to each plate.

### 2.8. Sample Solution Preparation

Food products were purchased from local supermarkets and classified as peanut products or non-peanut products. The solid products were crushed and protein content was extracted with 1:10 (w/v) PBS containing 2% Tween and 1 M NaCl for 2.5 h at room temperature. The extract was centrifuged at 8,000 rpm for 10 min and the supernatant was stored at 4 °C. The sample solutions were diluted with 1:10 (w/v) 0.01 M PBS and used immediately.

### 2.9. Spiking and Recovery Experiments

Pure milk with 6% lipid content though high pressure sterilization was diluted with 1:10 (w/v) 0.01 M PBS and spiked with Ara h 1 at 60, 120, and 240 ng/mL. The Ara h 1 concentration in the spiked sample was calculated based on a standard curve of the developed sandwich ELISA.

### 2.10. Cross-Reactivity Determination

Cross-reactivities for a second major peanut allergen (Ara h 2), Ara h 3, BSA, egg albumin (OVA), soy protein, and pea protein were determined [17].

## 3. Results and Discussion

### 3.1. Production of Anti-Arah1 mAbs

Sodium dodecyl sulfate-polyacrylamide gel electrophoresis revealed a 63-kDa band for purified Ara h 1 for use as an immunogen. The mouse with the highest titer via ELISA was selected for cell fusion. Seven days after cell fusion, hybridoma cell growth was observed and single Sp2/0 murine myeloma and spleen cells were removed from the 96-well cell culture plates. The cell supernatant from the microplate wells was tested for anti-Ara h 1 activity by indirect ELISA. Cells strains with high affinity for Ara h 1 were selected and subcloned three times using a limiting dilution method. Consequently, 12 cell strains with high affinity for Ara h 1 were obtained and injected into the mice. mAb 1 to mAb 12 from ascitic fluid were recovered for mAb preparation.

### 3.2. Pairwise Interaction Analysis of the Ara h 1 mAbs

The affinities of the HRP-labelled mAbs were tested using the sandwich ELISA. The highest P/N value was obtained using mAb 6 as the capture antibody and HRP-conjugated mAb 4 as the detection antibody by sandwich ELISA (Table 1). Therefore, this combination was selected for subsequent experiments.

**Table 1:** Sandwich ELISA for pairwise interaction analysis (P/N value).

DetectionmAb	Capture mAb											
	1	2	3	4	5	6	7	8	9	10	11	12
1-HRP		1.14	0.49	1.21	1.47	4.29	1.42	2.00	1.05	1.21	0.90	0.87
2-HRP	1.16		0.65	0.85	0.96	1.11	0.98	1.02	1.45	1.07	1.16	0.94
3-HRP	0.94	1.71		0.65	0.98	2.05	0.75	1.22	1.76	1.40	2.51	1.21
4-HRP	1.10	1.00	0.59		0.72	7.04	1.16	0.93	1.64	0.91	1.02	1.24
5-HRP	0.81	0.59	1.09	0.81		0.79	0.73	0.99	0.49	0.71	0.65	0.84
6-HRP	0.99	1.43	0.91	0.51	0.87		1.01	1.01	0.29	1.10	0.90	1.26
7-HRP	1.37	0.97	1.07	1.07	1.10	1.14		1.37	1.40	1.34	1.44	2.80
8-HRP	1.07	1.61	0.94	0.83	0.99	0.95	0.97		1.13	0.92	0.89	0.97
9-HRP	0.79	0.66	1.03	1.03	1.14	1.26	1.15	0.86		0.90	1.04	1.26
10-HRP	1.09	0.83	1.61	1.05	0.59	0.67	1.35	1.04	0.96		1.02	0.94
11-HRP	0.99	0.63	0.62	0.80	0.93	0.62	1.05	0.58	1.12	0.99		0.63
12-HRP	0.98	0.19	0.86	1.37	1.00	1.27	2.94	0.94	0.74	1.28	1.14	

Note: P/N value was the OD450 ratios of the positive and negative controls.

### 3.3. Development of an mAb-based Sandwich ELISA for Ara h 1 Detection

**Figure 1:**
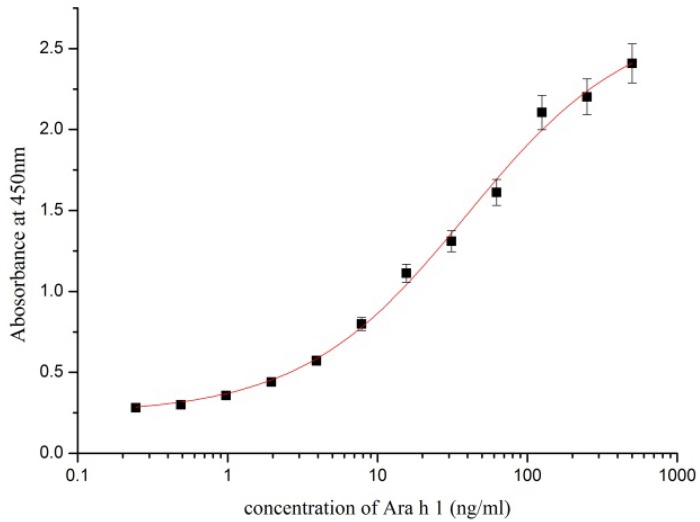
Calibration curve of the optimised sandwich ELISA.

The sandwich ELISA was established for Ara h 1 detection using the six capture mAbs at 2 µg/mL and four HRP-conjugated mAbs at 1:800 dilution for detection. Commercially purified ST-AH1 prepared in 0.01 M PBS was used as a standard (Figure 1).

**Figure 2:**
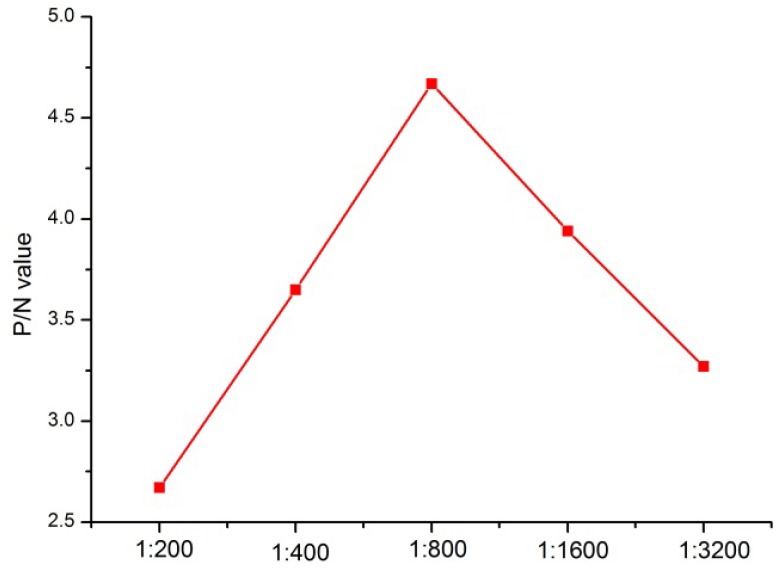
The optimization of the concentration of HRP-conjugated mAb 4.

The optimal dilution (1:800) with the highest P/N value was obtained using serially diluted mAb 6 as capture antibody and mAb 4 as detection antibody (Figure 2). The limit of detection of Ara h 1 was determined to be 0.34 ng/mL, which was obtained by the average baseline value plus 3 × SD, using functions available [18].

### 3.4. Recovery and Validation Studies

The results of inter- and intra-assay validation using Ara h 1-spiked and -free pure milk for the developed ELISA are shown in Table 2. The intra-assay recovery ranged from 96.62% to 106.23% and the inter-assay recovery from 95.45% to 105.18%. The intra- and inter-assay coefficients of variation ranged from 4.12% to 6.85% and from 4.87% to 12.35%, respectively. These data showed that this method produced accurate and repeatable results.

**Table 2:** Recovery of Arah1 from spiked pure Milk.

Spiked level (ng/mL)	Intra-assay (n = 6)		Inter-assay (n = 6)	
	Mean ± SD (ng/mL)	Recoveries (%)	Mean ± SD (ng/mL)	Recoveries (%)
60	58.04 ± 0.31	96.62	57.39 ± 2.14	95.45
120	127.98 ± 3.72	106.23	126.14 ± 4.88	104.87
240	245.34 ± 9.15	102.18	253.11 ± 12.81	105.18

### 3.5. Detection of Ara h 1 in Food

Ten products classified into peanut products and non-peanut products were analyzed using the optimized ELISA, and the OD_450_ ratios of the food samples (S) and the blank value (N) were compared with the value 2.1 to determine whether the foods were positive or negative for the Ara h 1 antigen. Seven foods (S/N ratio > 2.1) showed positive results, whereas three foods (S/N < 2.1) showed negative results (Table 3).

**Table 3:** Detection of Arah1 in the commercial foods.

Samples	Results	ELISA mean (ng/g)
Peanut butter(sijibao)	Positive	>50,000
Peanut cookies(kangshifu)	Positive	>50,000
Peanut chocolate bars	Positive	478
Roasted peanut(wangwang)	Positive	7,004
Roasted peanut(koushuiwa)	Positive	7,281
Red dates cake	Negative	<LOD
Milk containing peanut(yinlu)	Positive	>50,000
Milk containing peanut(hao+1)	Positive	>50,000
Pure milk(yili)	Negative	<LOD
Roasted melon seeds(alishan)	Negative	<LOD
Soybean oil(gold arowana)	Negative	<LOD

### 3.6. Cross-Reactivity Determination

**Figure 3:**
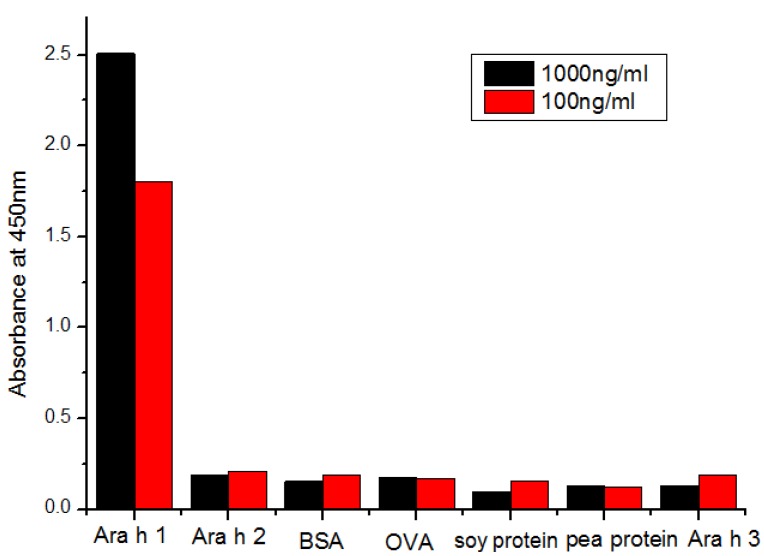
The cross-reactivity determination results with the similar proteins.

The results showed no cross-reactions with Ara h 2, BSA, OVA, soy protein, and pea protein, Ara h 3 (Figure 3).

## 4. Conclusions

The peanut is one of the most frequent causes of food allergies and Ara h 1, a major peanut allergen, can be used as marker for peanut content in foods. Thus, we have developed a highly sensitive and specific monoclonal anti-body-based ELISA to monitor Ara h 1 content in foods. For development of the proposed assay, 12 mAbs with high affinity and specificity were obtained and labelled with HRP. Through pairwise interaction analysis, the combination of six capture mAb and four HRP-mAb for optimal binding and sensitivity of the developed sandwich ELISA method revealed a sensitivity of 0.34 ng/mL. Compared with other assays, the sandwich ELISA we established increased sensitivity nearly a hundred-fold which has momentous significance in highly sensitive detection, especially in China's domestic product testing market.

Spiking and recovery experiments showed that the method was highly accurate, reliable, and reproducible. Furthermore, in the established ELISA, all seven types of commercial foods containing peanuts showed positive results and each of three types of non-peanut-containing products showed negative results, indicating a highly sensitive and specific sandwich ELISA to monitor Ara h 1 levels in foods to estimate potential exposure to peanut allergens.
